# Giant panda age recognition based on a facial image deep learning system

**DOI:** 10.1002/ece3.9507

**Published:** 2022-12-04

**Authors:** Yu Qi, Han Su, Rong Hou, Hangxing Zang, Peng Liu, Mengnan He, Ping Xu, Zhihe Zhang, Peng Chen

**Affiliations:** ^1^ School of Computer Science Sichuan Normal University Chengdu China; ^2^ Visual Computing and Virtual Reality Key Laboratory of Sichuan Province Chengdu China; ^3^ The Third Institute of Photogrammetry and Remote Sensing Ministry of Natural Resources Chengdu China; ^4^ Chengdu Research Base of Giant Panda Breeding Sichuan Key Laboratory of Conservation Biology for Endangered Wildlife Chengdu China; ^5^ Sichuan Academy of Giant Panda Chengdu China; ^6^ Giant Panda National Park Chengdu Administration Chengdu China

**Keywords:** age classification, convolutional neural network, deep learning, giant panda, wildlife ecology

## Abstract

The conservation of the giant panda (*Ailuropoda melanoleuca*), as an iconic vulnerable species, has received great attention in the past few decades. As an important part of the giant panda population survey, the age distribution of giant pandas can not only provide useful instruction but also verify the effectiveness of conservation measures. The current methods for determining the age groups of giant pandas are mainly based on the size and length of giant panda feces and the bite value of intact bamboo in the feces, or in the case of a skeleton, through the wear of molars and the growth line of teeth. These methods have certain flaws that limit their applications. In this study, we developed a deep learning method to study age group classification based on facial images of captive giant pandas and achieved an accuracy of 85.99% on EfficientNet. The experimental results show that the faces of giant pandas contain some age information, which mainly concentrated between the eyes of giant pandas. In addition, the results also indicate that it is feasible to identify the age groups of giant pandas through the analysis of facial images.

## Graphical abstract

In this work, a deep learning method was developed to study the distinctiveness of panda faces for age group classification. The experimental results show that the face of the giant panda contains some age information, and it is feasible to identify the approximate age group of the giant panda through the analysis of facial images.

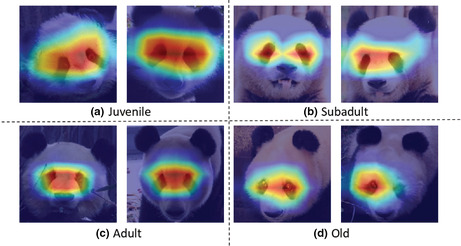

## 1 INTRODUCTION

As a rare and vulnerable animal endemic to China and a flagship species for wildlife conservation, the giant panda has an extremely high research value and important conservation significance (Zhang et al., 2007). The giant panda is considered an umbrella species as the conservation strategies to protect it and its habitat benefits sympatric species, such as the takin (*Budorcas taxicolor*), golden snub‐nosed monkey (*Rhinopithecus roxellana*), crested ibis (*Nipponia nippon*), dwarf musk deer (*Moschus berezovskii*), red panda (*Ailurus fulgens*), and blood pheasant (*Ithaginis cruentus*) (Li & Pimm, 2016). In addition to wildlife, giant panda conservation also protects the ecosystem functions that are closely related to the survival of hundreds of millions of people in the Yangze river basin (Kang et al., 2013; Pimm et al., 2018; Wang et al., 2022; Wei et al., 2018).

To conserve giant pandas in the wild, the Chinese government and international organizations, such as World Wide Fund for Nature (WWF), have carried out numerous studies on the population structure using several census methods including route survey, biological population horizontal density, distance discrimination, and distance‐bite discrimination (Tang et al., 2015). At present, according to the results of the fourth national survey of giant pandas completed in 2014, the population of wild giant pandas has grown steadily, the habitat has been significantly expanded, and the conservation and management capabilities have gradually increased compared with the results of the previous three surveys (Tang et al., 2015). As a result of these improvements, the giant panda was downgraded from “endangered” to “vulnerable” by the International Union for the Conservation of Nature (IUCN) in 2017 (Swaisgood et al., 2016). However, despite significant conservation management success, fragmentation of the giant panda habitat remains a major threat to its survival, and some local populations still face survival risks. Furthermore, despite improvements in conservation methods, all previous national surveys have failed to obtain a comprehensive and accurate age structure of the wild panda population (Tang et al., 2015). Understanding the age structure of a population, particularly small, isolated populations, is very important for understanding the stability and reproductive potential of that population. As different age groups have a large impact on the birth rate and mortality of a population, research on the age distribution of different giant panda populations can monitor and even predict population dynamics in real‐time (Gong et al., 2012).

In recent years, age estimation, as an emerging biometric recognition technique, has become a research focus in computer vision. It has been widely used in areas such as age‐based human‐computer interaction, commercial law enforcement, and biometrics (Jain et al., 2004; Panis et al., 2016; Pinter et al., 2017; Raval & Shankar, 2015). Usually, age estimation is considered a multi‐classification or regression problem. In the early face age estimation studies, most of the studies used hand‐designed face age‐related features (Guo et al., 2009; Lowe, 1999) and then estimated face age by classification or regression models. However, the design of features and the choice of learning methods often require rich prior knowledge, which deeply affects the performance of age estimation. In recent years, deep convolutional neural networks (CNN) have gained importance in the field of machine learning and pattern recognition due to their good feature extraction capabilities. Several works started to experiment with deep learning models for age classification or regression. Levi and Hassncer (2015) learn representations by deep convolutional neural networks for automatic age and gender classification. Niu et al. (2016) treat age estimation as an ordered regression problem and solve the ordinal regression problem by an end‐to‐end learning method using CNN. Zhang et al. (2019) investigate the limitations of small‐scale image compression models and propose a compact and efficient context‐based cascaded age estimation model (C3AE). Zang et al. (2021) propose the LERep model for age estimation to solve the model for applications on low‐power devices. With the development of computer technology, researchers in the field of animal biometric recognition have started to conduct research on species recognition based on phenotypic appearance using computer technology (Kühl & Burghardt, 2013; Polzounov et al., 2016; Gomez Villa et al., 2017; Norouzzadeh et al., 2018; Li et al., 2019). Similarly, methods used for face recognition with humans have been applied to wildlife. For example, in terms of individual recognition, Hou et al. (2019) proposed to recognize the face of giant pandas based on the developmental network. Later, Matkowski et al. (2019) proposed a panda facial recognition algorithm. Chen et al. (2020) and Hou et al. (2020), respectively, proposed a recognition framework and method based on deep learning and achieved a good recognition effect in their respective datasets of captive pandas. While He et al. (2019) proposed a deep learning model for red panda recognition and Schofield et al. (2019) presented a CNN method for face detection, tracking, and recognition of wild chimpanzees from long‐term video records. Finally, Guo et al. (2020) designed a deep neural network with an attention mechanism for individual identification of primates and other species, achieving an accuracy of 91.1%–97.7%. In terms of gender recognition, Wang et al. (2019) proposed a gender classification method for giant pandas based on a deep neural network and achieved an accuracy rate of 77.2%, providing a basis for the field of gender classification research. These studies provide evidence that mammalian facial features contain important information needed for individual identification (Kumar et al., 2017).

Currently, the method of classification for different age groups of giant pandas is still mainly based on biological methods, such as the size of giant panda feces and the length of intact bamboo found in feces, which is used to determine the different bite values for giant pandas (Hu, 2011). Additionally, the wear of molars and the growth line of teeth may be used to determine the age of a giant panda skeleton; however, this is not useful for determining the current age structure of a population (Wei et al., 2011). Although these methods of estimating the age groups of giant pandas are simple, they are impractical because they require a significant amount of difficult fieldwork to collect fecal samples, which is time‐consuming and labor‐intensive as well as having high‐risk factors, high collection costs, and poor overall accuracy (Zhan et al., 2009). Therefore, new objective and reliable techniques are needed to effectively estimate the age groups of giant pandas.

To conserve and monitor giant pandas, relevant government agencies have deployed a large number of infrared cameras in the wild, which provides a foundation for noninvasive monitoring systems. With the development of image processing technology, the improvement of the quality of image acquisition equipment, and the upgrading of image transmission methods, image‐based age group recognition technology for giant pandas have become possible. In addition, the data used for age group classification are low‐cost, noninvasive, safe, easy to collect, and not easily affected by natural factors. Therefore, this technology may solve the limitations of the age group classification and population structure of wild populations of giant pandas. In this paper, a deep learning method was developed to study the distinguishability of giant panda faces for age group classification. The experimental results show that giant pandas' faces contain some age information, and it is feasible to identify the age group of giant pandas by analyzing facial images. To our knowledge, there is no precedent for estimating the age groups of giant pandas. It is important to note that the availability of data captured by field cameras is limited. This paper mainly introduces the dataset acquired from captive pandas. This noninvasive approach is expected to provide technical support for the giant panda surveys and population monitoring.

## 2 MATERIALS AND METHODS

### 2.1 Data collection

**FIGURE 1:**
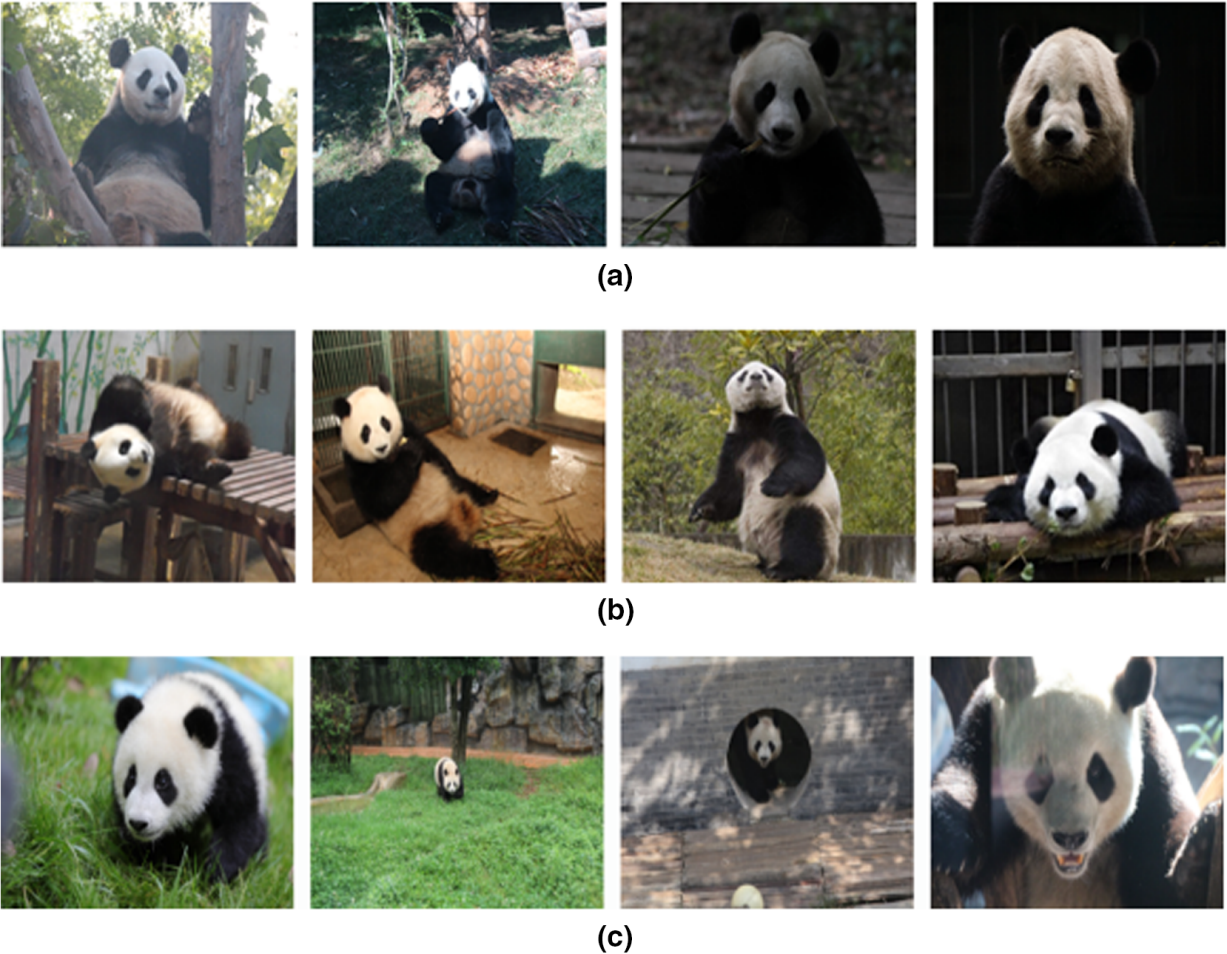
Examples of datasets. (a): Samples with different light and shade; (b): samples with different poses; (c): samples with different vision and scales.

By using a Panasonic dvx200 video camera and three digital cameras (Canon 1DXmarkII, Canon 5DmarkIII, and a Panasonic Lumix DMC‐GH4), the image data of 218 captive pandas were collected. The image dataset of captive giant pandas used in this study are all from the Chengdu Research Base of Giant Panda Breeding (164 individuals), the China Conservation and Research Centre for the Giant Panda (37 individuals), the Beijing zoo (7 individuals), the Research center for the Qinling giant panda (5 individuals), the Chongqing zoo (3 individuals), the Chengdu zoo (1 individual) and the Fuzhou zoo (1 individual). All data were cleaned and annotated to establish a larger giant panda age image database. Detailed information about each individual, such as a family tree, date of birth, sex, and reproductive history is strictly managed and recorded by the National Forestry and Grassland Administration of China and the Chinese Association of Zoological Gardens. Due to the conservation status and seclusive behavior of giant pandas, images can only be collected by specialized trained field staff. The processing of collected images is also very difficult, and there are usually three problems:

1. The captured image data have different lighting, due to the wide data sources and the influence of weather changes (Figure 1a)
2. The captured image data feature giant pandas in different positions. Although the diversity of poses is beneficial to the generalization ability of the model, it also increases the data requirements for various poses (Figure 1b).
3. The captured image data feature different shooting angles and scales. Generally, the photographers are collecting images from outside the animal enclosure. This creates issues because the complex environment within the animal enclosure limits the viewing angle and scale of the shooting, resulting in large differences in the size of the collected images, and some occluded images (Figure 1c).

In response to the above image collection problems, we screened the dataset. At the same time, for the convenience of defining uniform selection conditions, we chose the face of the giant panda as the focus of the study. Since faces contain many unique attributes and have been previously applied in facial age recognition (Antipov et al., 2017; Levi & Hassncer, 2015; Samek et al., 2017) and individual panda recognition studies (Chen et al., 2020; Hou et al., 2019, 2020).

**FIGURE 2:**
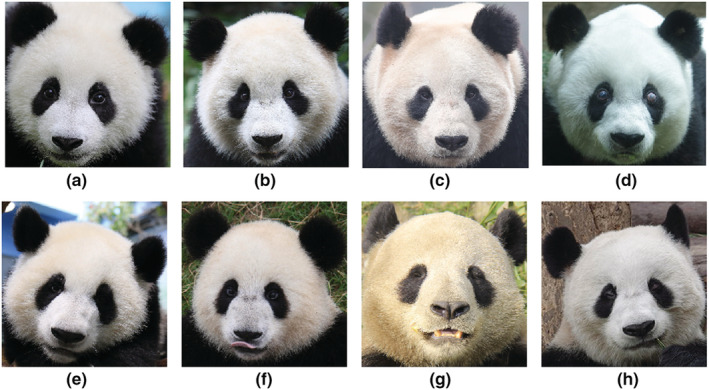
Samples from four age groups. Images in the first row are easy examples and in the second row are hard examples, (a) and (e) are juveniles, (b) and (f) are subadults, (c) and (g) are adults, (d) and (h) are elders.

We collected 6441 images of 218 pandas of different ages following Hu (2011), including 64 juvenile giant pandas (<1.5 years old) with 784 images, 97 subadult giant pandas (1.5–5.5 years old) with 1294 images, 121 adult giant pandas (5.5–20 years old) with 4035 images, and 14 old giant pandas (20–27 years old) with 328 images. Figure 2 shows samples from four age groups, simple samples that can be recognized by human vision and hard samples that may not be recognized. It is really hard to distinguish the age of giant pandas based on pictures for human vision, and it is challenging enough to estimate the age groups.

**FIGURE 3:**
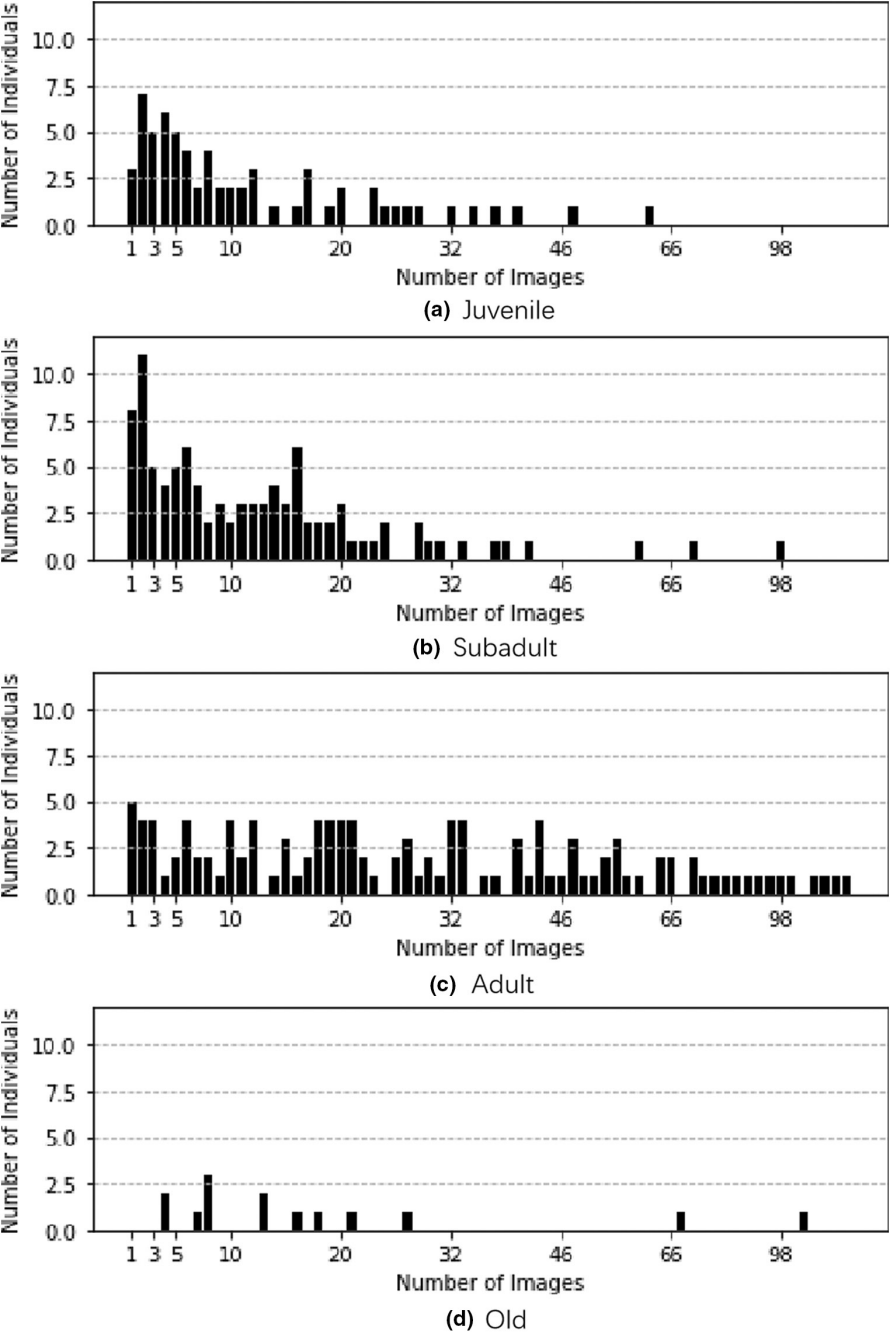
Distribution of individual data. The *X*‐axis is the number of images, and the *Y*‐axis is the number of individual pandas. (a), (b), (c), and (d) refer to histograms of each age group, respectively.

Figure 3 shows the number of images for each individual in each age group, where the *x*‐axis represents the number of images, and the *y*‐axis represents the number of individuals containing the corresponding number of images. It is worth noting that some individual data span multiple age groups.

### 2.2 Image annotation

**FIGURE 4:**
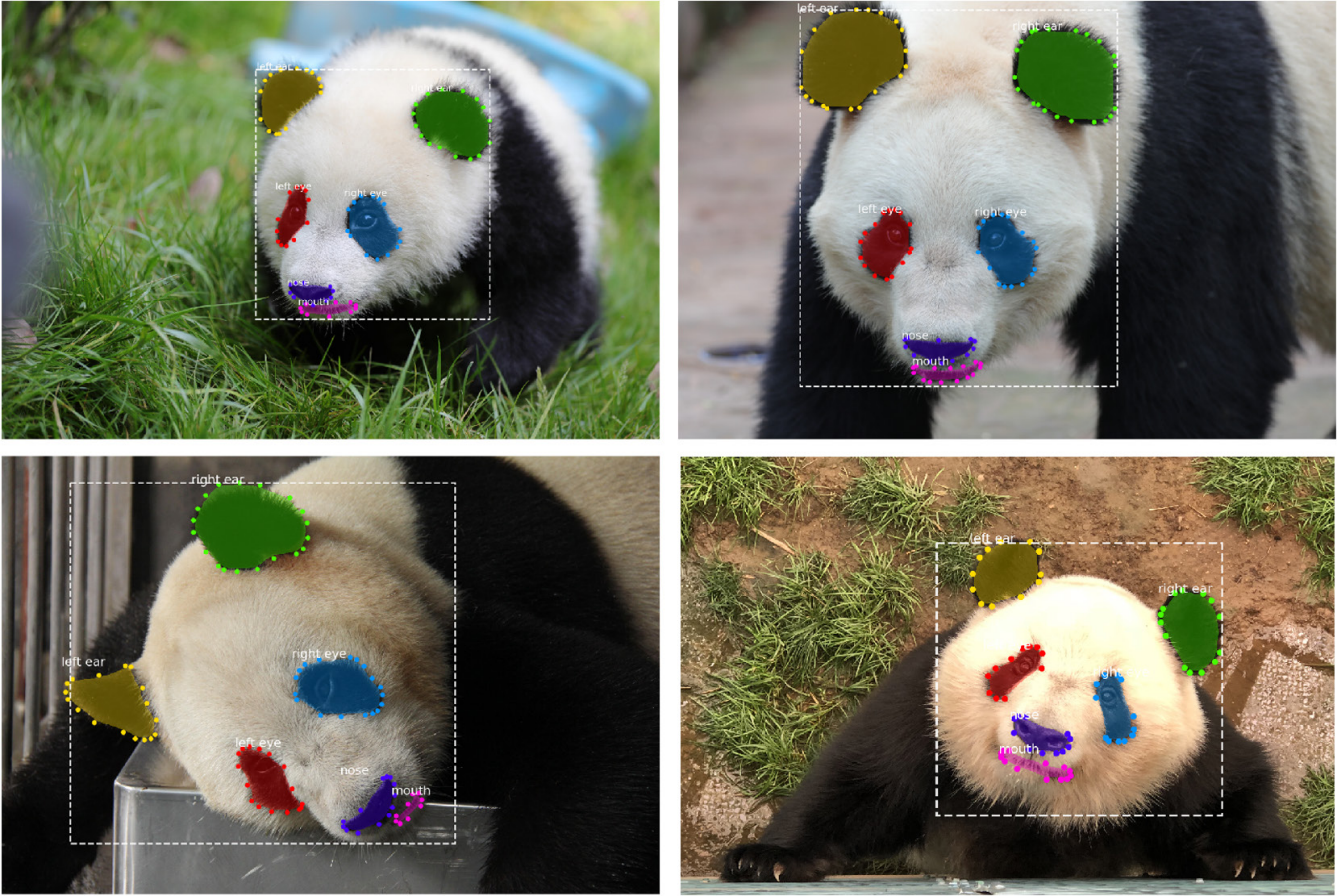
Examples of automatic annotating. The automatic annotation tool annotates the eyes, nose, ears, mouth, and the whole head of the giant panda, respectively.

Based on the background of the above‐mentioned dataset, we have performed regional annotations on the filtered and sorted images, which can be used for subsequent tasks such as target detection. The research area of this paper is the face of the giant panda, so the marked area is the facial features (i.e., ears, eyes, nose, and mouth) and face of the giant panda. Firstly, manual annotation was carried out through the VIA (Dutta & Zisserman, 2019) annotating tool. After the manual annotation of more than 400 panda images, fine‐tuning of mask‐RCNN(He et al., 2017), which was pretrained on a Microsoft COCO dataset (Lin et al., 2014), was started. Since the edges of the facial features of the giant panda are obvious, in the early stage of the automatic annotating model training, the model achieves a good automatic annotating effect and effectively improves the efficiency of regional annotating. Some examples of automatic labeling are shown in Figure 4. With the automatic annotation model, we were able to detect and segment the facial features and faces of giant pandas well, which provided effective facial image data for the subsequent prediction of giant panda age groups.

### 2.3 Network and architectures

Many pandas in different age groups have very similar appearances and cannot be easily distinguished by the human eye (Figure 2e–h). To learn the advanced features that may not be obvious in human vision, we needed to build a deep model to extract the features. For this, we used a Convolutional Neural Network (CNN; LeCun et al., 1998) and Residual Learning (He et al., 2016a).

For giant panda images, a deep neural network needed to be constructed to extract features at a deep level; however, the training of deep neural networks faced network degradation problems. Residual learning effectively solved this problem and can train neural networks with more than 1000 layers (He et al., 2016a). In the case that the network is getting deeper and deeper, the residual connection and identity mapping in the residual block (He et al., 2016b) can effectively avoid gradient disappearance and gradient explosion, and it is easier to optimize and improve the accuracy. Therefore, in this paper, ResNet (He et al., 2016a) was introduced as one of the experimental models for the age group classification of giant pandas. In addition, in order to further improve the accuracy and speed of the network, it is necessary to balance the three dimensions of width, depth, and resolution, EfficientNet (Tan & Le, 2019) was also introduced in this paper as an experimental model for the age group classification of giant pandas.

For this study, we used a modified ImageNet pretrained model to fit our tasks of age group classification workflow (Figure 6). The last fully connection layer of the experimental model was replaced by different numbers of neurons according to different tasks. According to the age group division of giant pandas, we set the age group classification of giant pandas as a multi‐classification problem, so there were four neurons connected to the fully connection layer of the feature extractor. The features were mapped to the scores of each category through the fully connected layer, the goal was to enlarge the score of corresponding categories, and the loss function was defined as:

(1) $$L_{\mathrm{ce}}=-\frac{1}{N}\sum_{k=1}^{N}\log\frac{e^{w_{j}^{T}x+b}}{\sum_{i=1}^{m}e^{w_{i}^{T}x+b}},$$

where N is the number of samples, m is the number of categories, and the subscript j represents the index position in the real class label. This loss function, known as the cross‐entropy loss function, calculates the resulting loss by comparing the predicted probability of a category with the true value after the model has produced a predicted value, and then sets a penalty term in logarithmic form based on this loss. When training the model, the cross‐entropy loss function is used to minimize the loss, i.e., the smaller the loss the better the model. When we use this loss, we will train a CNN to output a probability over the m classes for each image. It is used for multi‐class classification.

**FIGURE 5:**
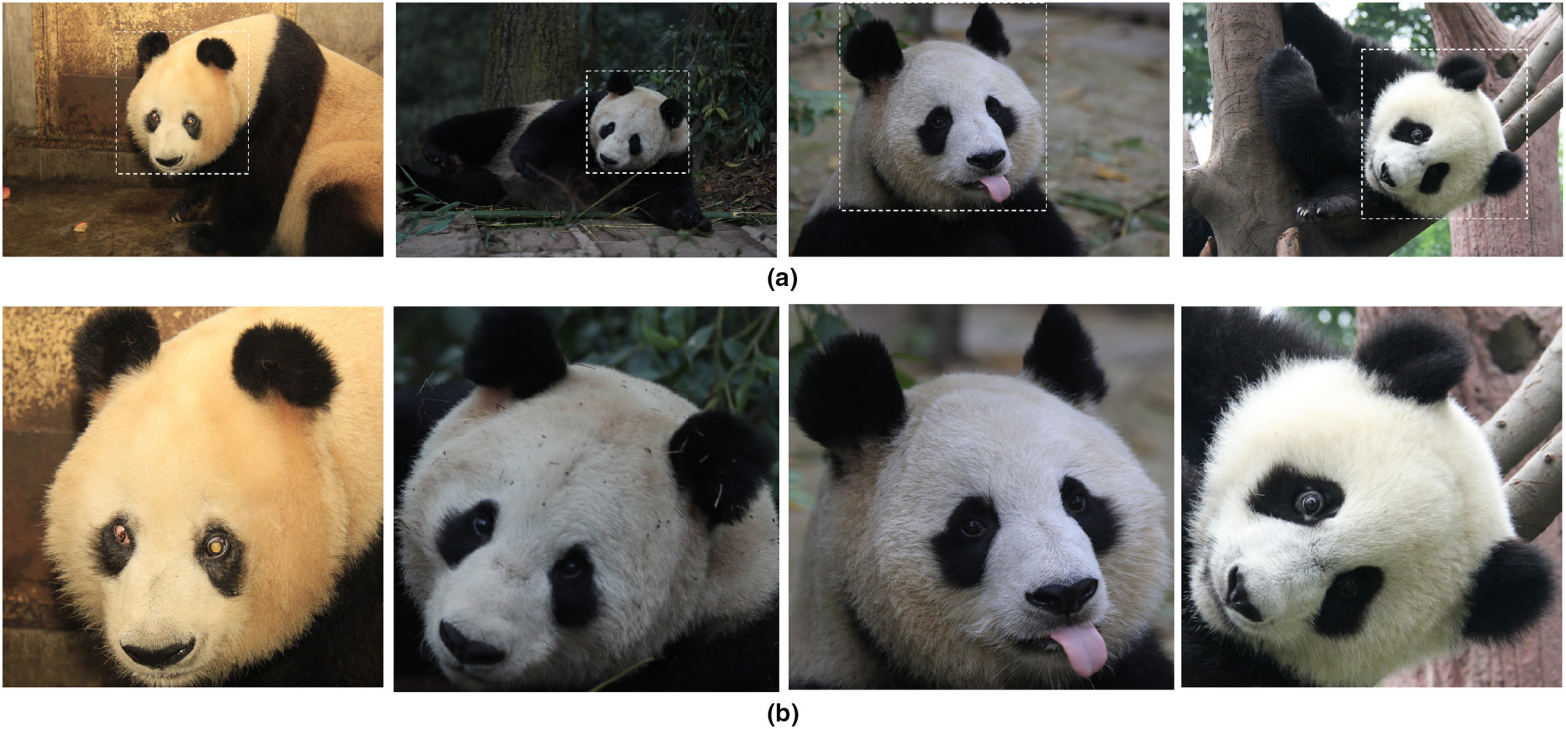
Examples of cropped images. (a): original samples; (b): cropped samples.

As the focus of this study was giant panda facial images, in order to verify the feasibility of the deep learning technology, we first crop the face according to the data annotation (Figure 5). The purpose was to reduce the influence of the background and let the model pay more attention to the learning of the foreground.

**FIGURE 6:**
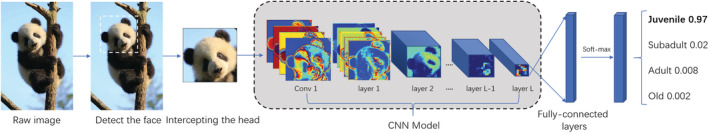
The age group classification workflow.

As shown in Figure 6, we first need to process the original image of the giant panda, i.e., truncate the face region of the giant panda (Figure 5), then input the face of the giant panda into the CNN model, and finally output our prediction results.

### 2.4 Model training

**FIGURE 7:**
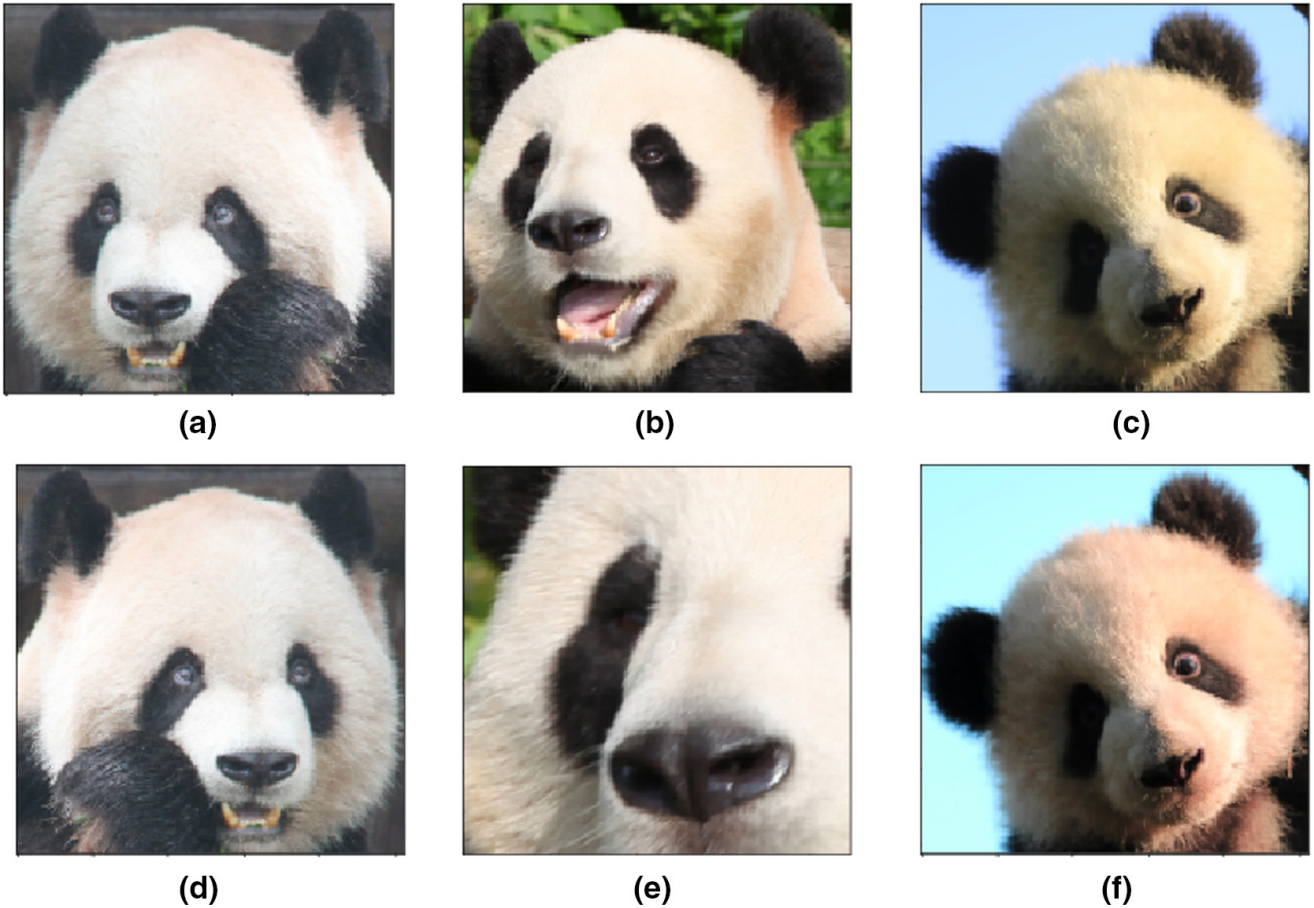
Example of raw and augmentation images. The first row is raw images, and the second row is augmentation images.

Since some individual data span multiple age groups, i.e., images of the same panda can appear multiple times, but at different ages and facial image features contain individual information (Kumar et al., 2017). To eliminate the interference of individual information, we first selected the images of this part of the individual data existing in multiple age groups as the training set. Then, we calculated the number of remaining individual images according to age groups and selected the individual image data with the number of images ranging from 10 to 30 as the test set, while the remaining images are used as the training set. The results of the dataset division are shown in Table 1. Although the current dataset is the largest panda face dataset, it is still small compared with training for deep learning methods. To alleviate this problem, we used data augmentation in the training set, including random cropping, random flipping, and random changes in image brightness, contrast, and saturation (Figure 7). In addition, we tried to use Gaussian noise and random erasure operations to improve the model generalization, but through experiments, we found that these two data augmentation methods did not improve the model performance, so we did not use these two methods. Images in the dataset are frontal face regions of giant pandas, which were manually cropped and resized to 512 × 512 pixels, resized to 256 × 256, and randomly cropped to 224 × 224 to fit the input of the model during the training phase.

**TABLE 1:** Statistical table of experimental data

Age groups	Training set		Testing set	
	Individuals	Images	Individuals	Images
Juvenile	60	679	5	105
Subadult	87	1130	10	164
Adult	92	3485	29	550
Old	11	276	3	52

Since there were no previous studies on the age of giant pandas based on facial images, to evaluate the performance of the giant panda age group classification framework, we selected some basic and representative network models for experiments, respectively, support vector machines (SVM) with linear kernel, VGG16 with BN (Simonyan & Zisserman, 2014), ResNet with different depths (ResNet18, ResNet34, ResNet50) and EfficientNet‐B0 (Tan & Le, 2019). For the SVM classifier, we first perform feature extraction on the giant panda image. Here we use the Histogram of Oriented Gradients (HOG) feature extraction (Dadi & Pillutla, 2016), which is a feature‐based descriptor used in image processing and computer vision for detecting objects, then using the SVM classifier to classify its age group. The VGG model has won first and second place in the ImageNet localization and classification competitions, respectively, and achieved very good results. The network structure of VGG is characterized as follows: (i) simple structure. The network consists of five convolutional and maximum pooling layers, three fully connected layers, and softmax layers, and uses the ReLU nonlinear activation function; (ii) small convolutional kernels. It has been concluded that the receptive field of two small convolutional kernels is equivalent to that of one convolutional kernel, while the receptive field of three small convolutional kernels is equivalent to that of one convolutional kernel. Therefore, VGG uses multiple small convolutional kernels instead of the large convolutional kernels in previous convolutional neural networks. This reduces the network parameters, decreases the complexity of the model, and increases the number of activation functions so that the network model can be better used for feature extraction.

The above‐mentioned network model weights based on deep learning are initialized as ImageNet pretraining weights, and all model output units are replaced by softmax output units with an output of 4. All our experiments were performed on a 64‐bit Ubuntu 16.04 computer, with an Intel E5‐2698 2.20GHz CPU and an NVIDIA Tesla V100 GPU. The model was implemented with Python 3.7 and PyTorch 1.10.0. The optimizer plays a crucial role in determining how quickly our model reaches its global minimum and the net loss our loss function generates at the end of the training phase. Currently, commonly used optimizers include SGD, Adam, RMSProp, and so on (Ruder, 2016). SGD is widely used by researchers around the world because it is good at finding flat minima that are relevant to generalization. Therefore, in this paper, SGD with momentum of 0.9 was adopted to accelerate SGD in the relevant direction and dampen oscillations, the weight decay was set to 5e‐3. The initial learning rate was set to 1e‐2 for EfficientNet‐B0 and 2e‐3 for ResNet and decayed by 0.3 every 6 epochs, the total epochs were 30, and the batch size was 16. Except for the VGG, the learning rate was 1e‐2 and decayed by 0.1 every 6 epochs, and the weight decay was set to 3e‐3, the rest of the network configurations were consistent with the above. To ensure the reproducibility of the methods, we used the same random seed for all. In all experiments, we used Accuracy, Mean Absolute Error (MAE), and F1‐Score as experimental evaluation criteria.

Accuracy is defined as the number of samples that the model can correctly predict for a given test set as a proportion of the number of all predicted samples. Since this experiment is a multi‐classification problem, we compare the model‐predicted labels with the true labels, and if the label values are the same, the prediction is correct, and vice versa. Thus, the accuracy can be defined as:

(2) $$\text{accuracy}=\frac{1}{N}\sum_{i=1}^{N}I\left(y_{i}={\hat{y}}_{i}\right),$$

where N is the number of samples, $y_{i}$ is the true label, ${\hat{y}}_{i}$ is the predicted label, and $I\left(\cdot \right)$ is the indicator function, i.e., the output value is 1 when the input is true, otherwise the output value is 0.

Mean Absolute Error is used to measure the average absolute error between the predicted and true values, and a smaller MAE indicates a better model, which is defined as:

(3) $$\mathrm{MAE}=\frac{1}{N}\sum_{i=1}^{N}|y_{i}-{\hat{y}}_{i}|.$$

The F1‐Score combines the precision and recall of a classifier into a single metric by taking their harmonic mean. In the binary classification task, F1‐Score can be defined as:

(4) $$F1-\text{Score}=\frac{2\times \text{Precision}\times \text{Recall}}{\text{Precision}+\text{Recall}},$$

where Precision indicates the number of actual positive samples among those predicted to be positive, which is defined as:

(5) $$\text{Precision}\left(P\right)=\frac{\mathrm{TP}}{\mathrm{TP}+\mathrm{FP}}.$$

Recall indicates the proportion of samples that are positive that are judged to be positive, which is defined as:

(6) $$\text{Recall}\left(R\right)=\frac{\mathrm{TP}}{\mathrm{TP}+\mathrm{FN}},$$

where TP is the number of correct positive predictions; FP is the number of incorrect positive predictions; FN is the number of incorrect negative predictions.

Since this experiment is a multi‐classification task, we replace F1‐Score with Macro‐F1. It obtains the F1‐Score values of each category by calculating Precision and Recall for each category separately and then takes the average to obtain Macro‐F1.

## 3 RESULTS

All experiments were carried out on support vector machines (SVMs) with linear kernel, VGG16 with BN, ResNet with different depths, and EfficientNet‐B0. The accuracy of all learning‐based models was better than that of traditional models. Compared with EfficientNet‐B0 and ResNet50 the accuracy of VGG16 with BN was lower, which showed that the learning ability of the deep model was stronger and the residual learning was effective. In this experiment, EfficientNet‐B0 had the highest accuracy compared with the other models (Table 2), so the analysis that follows only shows the results for EfficientNet‐B0.

**TABLE 2:** Age classification results

Methods	Accuracy	MAE	F1‐score
HOG + SVM	69.00%	0.343	0.472
ResNet18	82.32%	0.183	0.654
ResNet34	82.55%	0.183	0.686
VGG16 + BN	84.86%	0.155	**0.687**
ResNet50	85.30%	0.158	0.678
EfficientNet‐B0	**85.99%**	**0.142**	0.685

*Note*: The best results are shown in bold font.

Receiver‐operating characteristic (ROC) curve was used in our experiments as a visualization tool for evaluating classification models. The horizontal coordinate is the false positive rate, which indicates the probability of misclassifying a negative case as a positive case, and the vertical coordinate is the true positive rate, which indicates the probability of correctly classifying a positive case as a positive case. The closer the ROC curve is to the diagonal, the less accurate the model is. Area Under Curve (AUC) is defined as the area under the ROC curve. The AUC value is equivalent to the probability that a randomly chosen positive example is ranked higher than a randomly chosen negative example. Since our experiment was a multi‐classification task, we replace AUC with a Macro‐average ROC curve area (Fawcett, 2006) just like Marco‐F1. The larger the area it represents, the higher the accuracy of the model.

**FIGURE 8:**
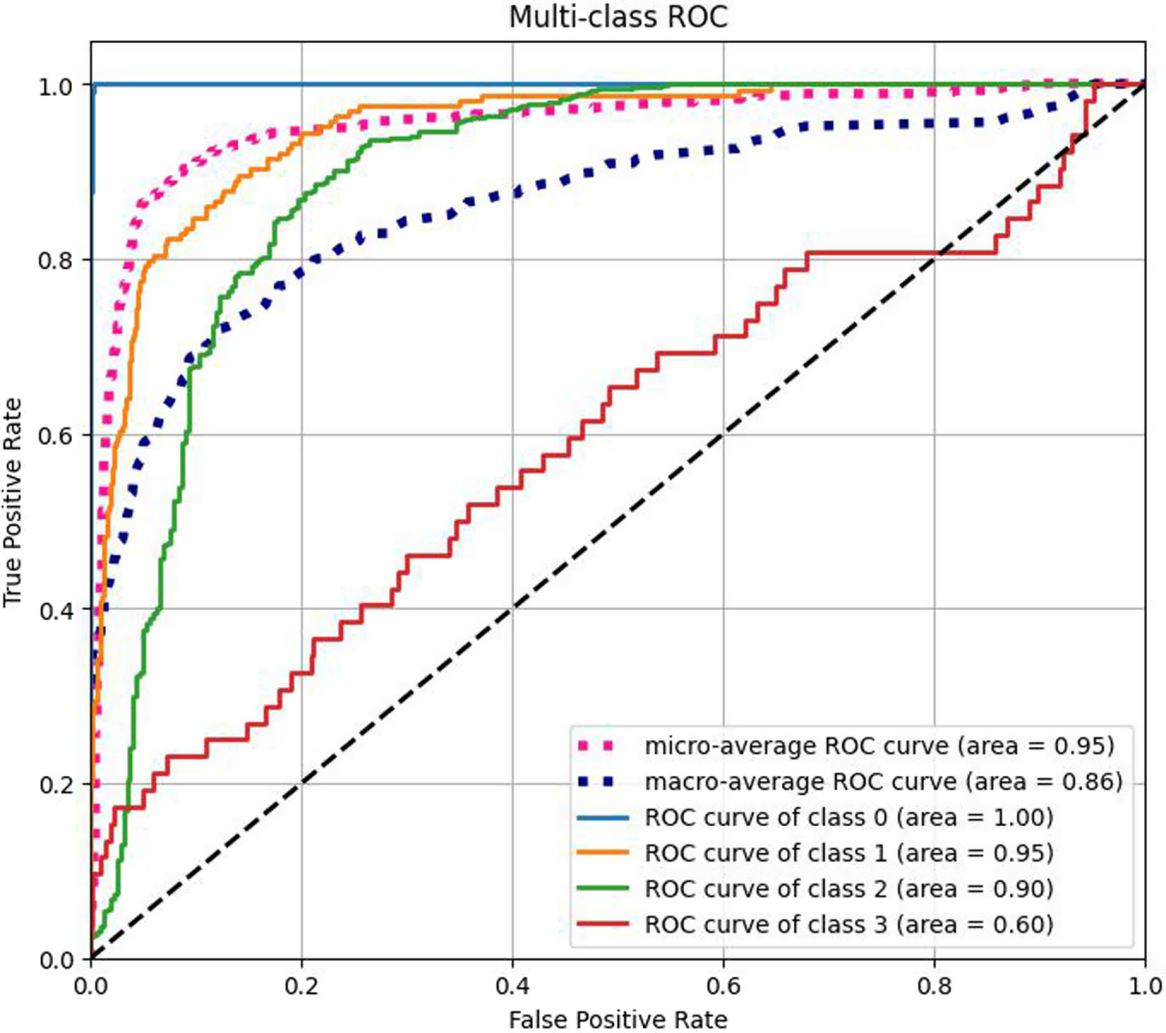
Receiver‐operating characteristic curve of EfficientNet‐B0.

The macro‐average ROC curves of EfficientNet‐B0 are given in Figure 8. The results showed that the macro‐average ROC curve area of the giant panda's age group classification was 0.86 indicating that the giant panda's face contains some age information, and it is feasible to classify the different age groups through facial recognition.

**FIGURE 9:**
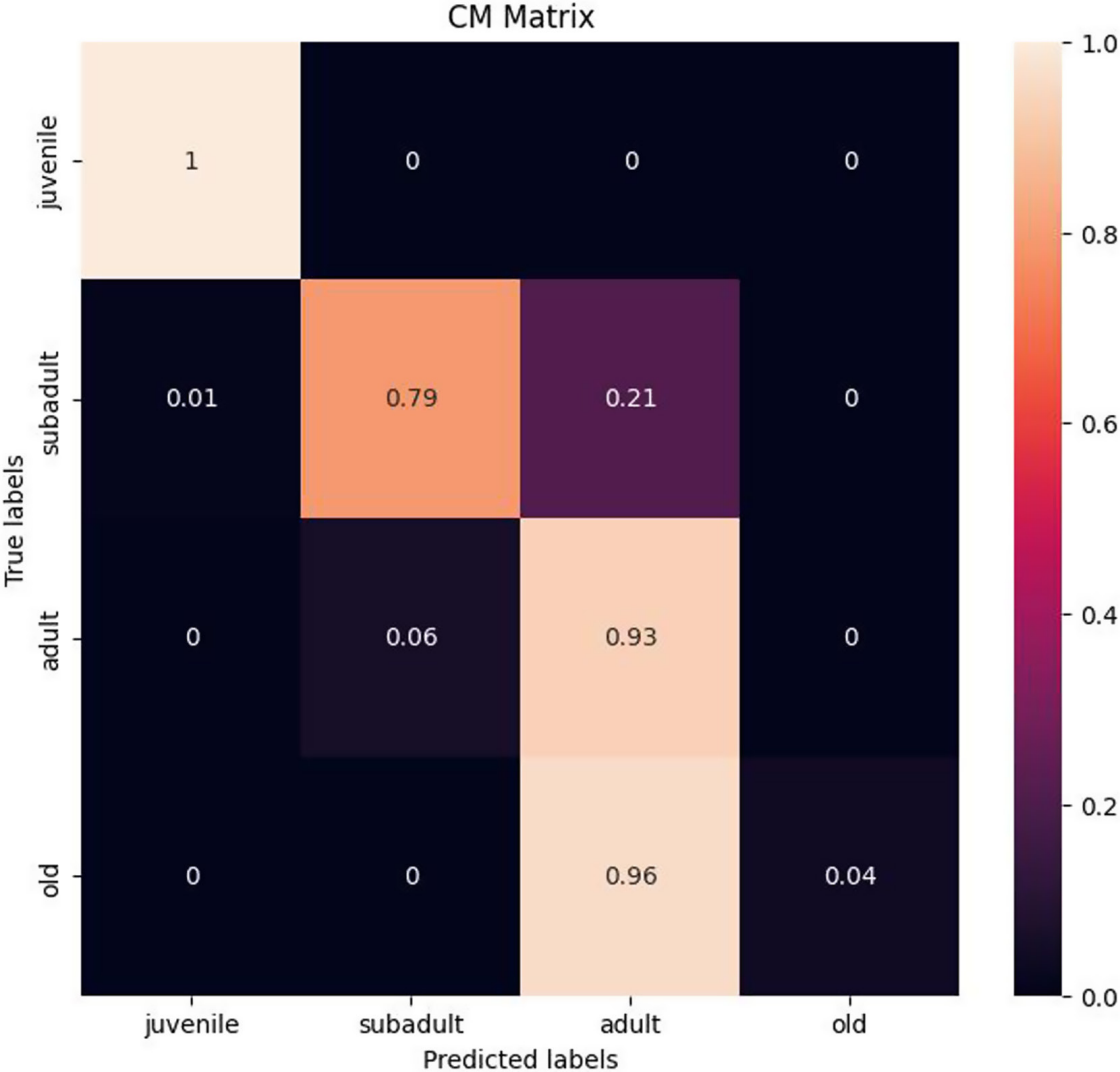
Confusion matrix of EfficientNet‐B0.

The model we employed had a better classification performance for the age group of juvenile and adult giant pandas, followed by the subadult group, and the weakest was in classifying the elderly panda age group (Figure 9). This may be since the fact that of the aging of giant pandas, their facial features gradually tend to be stable, making it difficult to distinguish the facial features of subadult from adult giant pandas, as well as adult and elderly giant pandas.

**FIGURE 10:**
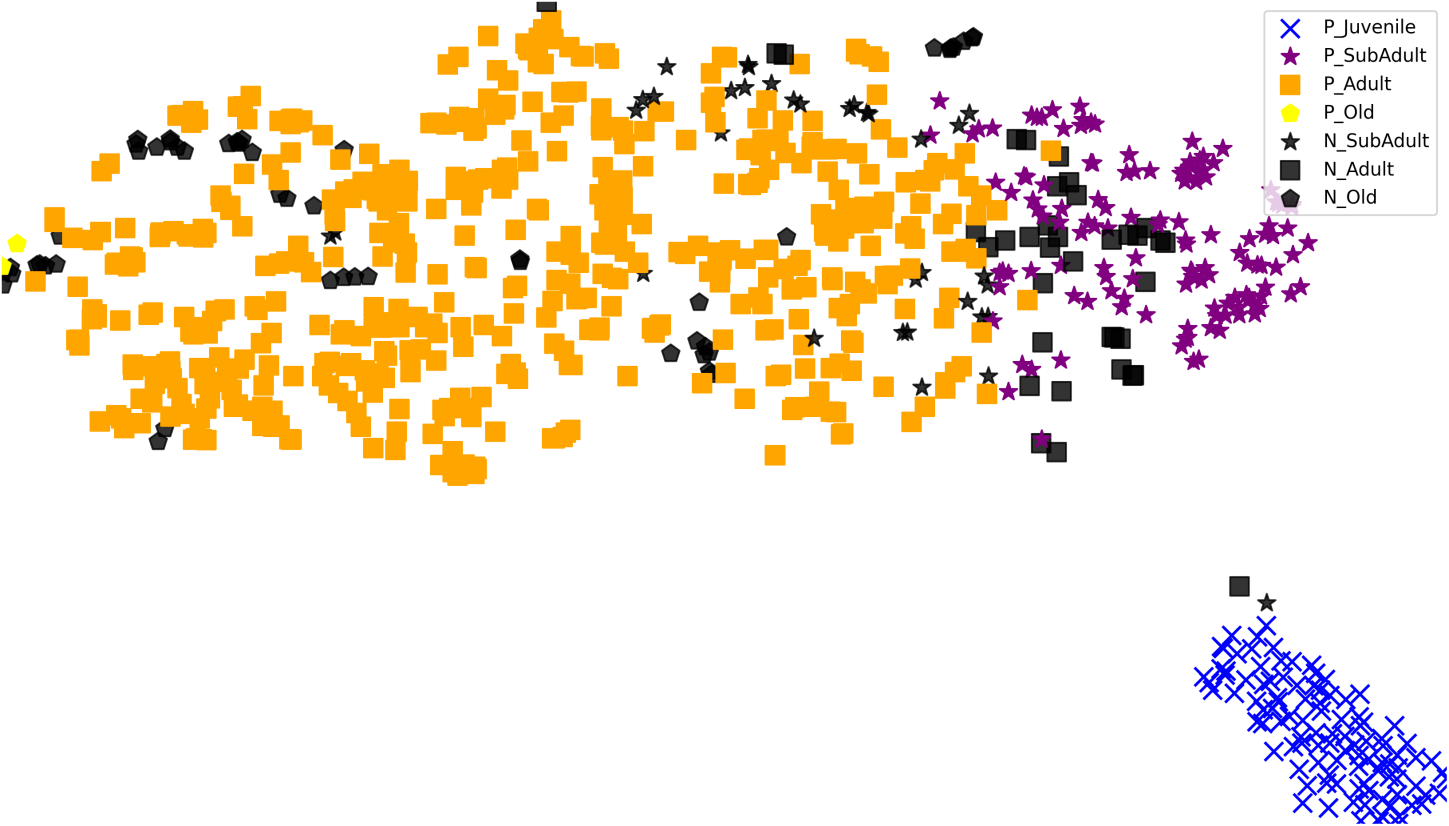
Visualization of the feature spaces defined by the feature learned by the proposed model. Black represents the negative samples, while other colors indicate positive samples, × represents juveniles, ★ represents subadults, ■ represents adults, and 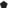 represents elders.

At the same time, a nonlinear dimensional reduction algorithm T‐SNE (T‐Distributed Neighbor Embedding) was adopted to visualize feature vectors (Van der Maaten & Hinton, 2008). From the feature visualization, after the model's learning of the data, it can distinguish the samples that were originally interactive and complicated in the feature space (Figure 10). Among them, juveniles, subadults, and adults had greater discrimination; however, the facial image features of the elderly giant panda were easily confused with those of adult pandas.

**FIGURE 11:**
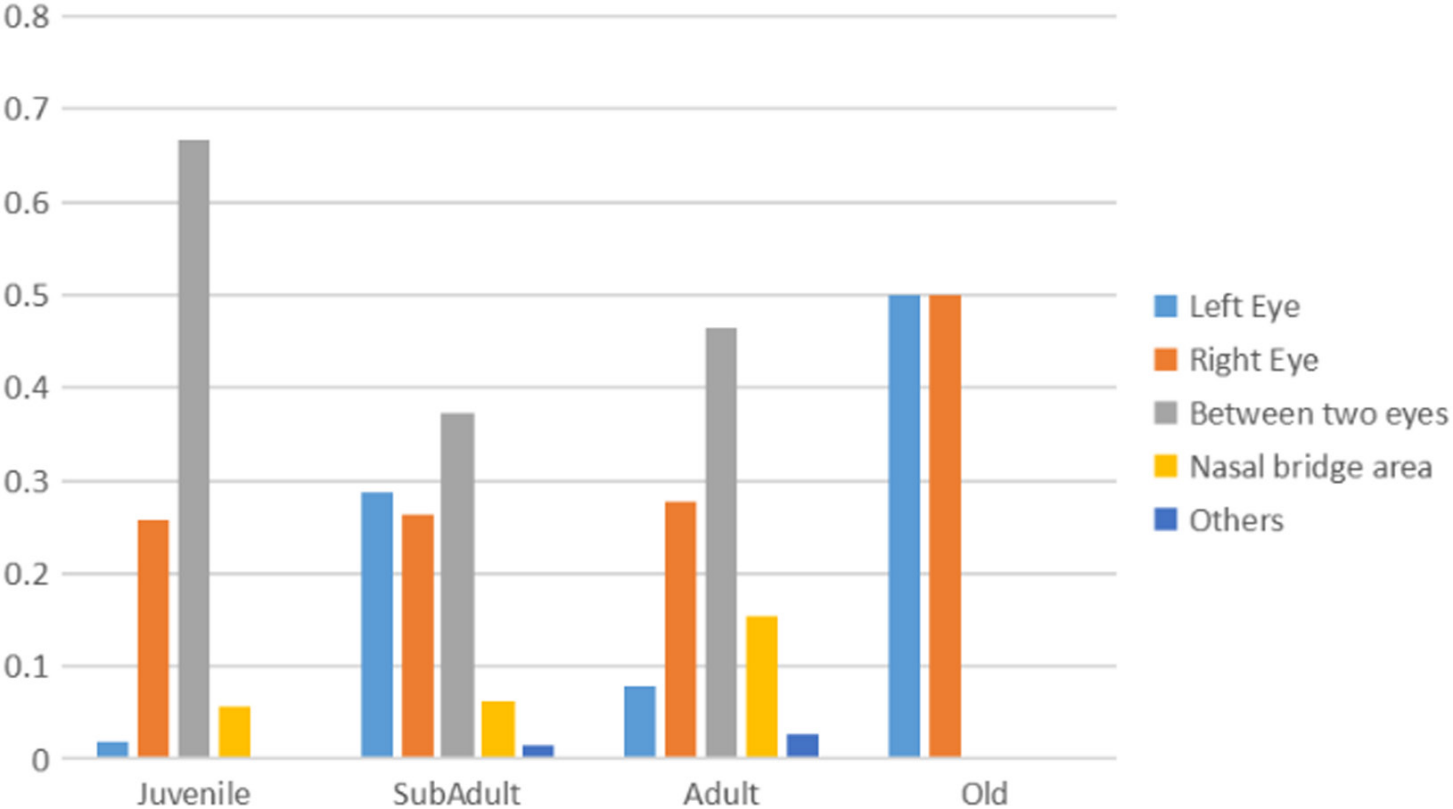
Quantitative results for the decision area. The decision area contains the left eye, the right eye, between the eyes, the nasal bridge area, and others.

**FIGURE 12:**
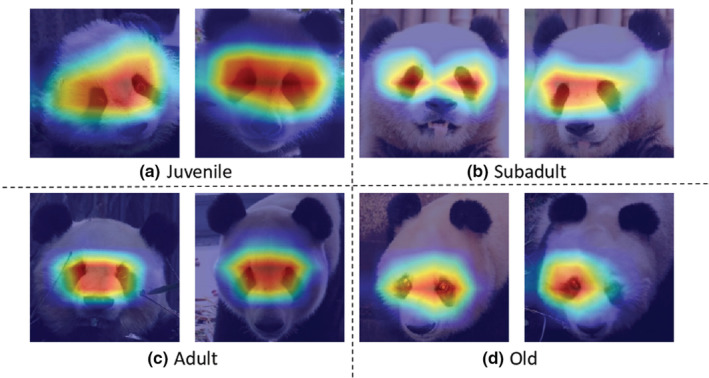
Visualization results via Grad‐CAM (a) is juvenile, (b) is subadult, (c) is adult, (d) is old.

In order to further explain and understand the model's focus on each age group, we employed the Grad‐CAM++ algorithm (Chattopadhay et al., 2018) on the final convolutional layer to localize and highlight the discriminative regions. Grad‐CAM++ is an upgrade of Grad‐CAM (Selvaraju et al., 2017), which is a class‐discriminative localization method. It assigns a score to each class using the backpropagation‐based filter gradient and convolution activation values. It provides a way to look into what particular parts of an image influence the model decision. In order to determine the decision region of the giant panda's age, we artificially classified and quantified the decision region of the panda's age into five categories: left eye, right eye, between two eyes, nasal bridge area, and others according to the output region of Grad‐CAM++ (Figure 11), and it can be seen that the decision region of the giant panda's age is mainly concentrated between the two eyes of the giant panda (Figure 12). These new findings will be further confirmed by biological and wildlife researchers.

## 4 DISCUSSION AND CONCLUSION

The age distribution of giant pandas is a key factor in understanding the population dynamics of the giant pandas in the wild, especially in the case of small, isolated populations that are at higher risk of local extinction (Wei et al., 2011; Xia & Hu, 2006). In this study, we explored the age group estimation of giant pandas in combination with computer image recognition technology. Our goal is to introduce cutting‐edge science, based on more accurate and efficient methods, to conduct auxiliary discriminant analysis of age structure, so as to contribute to the development of the in‐situ conservation and management of giant pandas. We used a deep convolutional network to learn features from the panda face image dataset, which contains 6441 images from 218 pandas. Our recognition approach by EfficientNet‐B0 can achieve 85.99% accuracy in estimating the age group of giant pandas based solely on facial images, which shows that this method of age recognition is feasible.

In order to find the distinguishable features of giant panda images by age, we visualized and quantified the facial features of the pandas (Figures 11 and 12) and found that the features learned by the proposed model are mainly concentrated in the triangular region of the eyes and nose of giant pandas, where the decision region is mainly concentrated between the two eyes of giant pandas. According to the visualization results, the faces of the giant pandas have certain age information, and the changes in facial features tend to stabilize as the giant panda matures gradually. Our results further reveal that, as the giant panda ages, the facial features become more distinct, and the distinguishable area changes from the triangle area between the eyes and nose of the giant panda to the area between the two eyes.

In this experiment, we found that while the classification accuracy of juvenile, subadult, and adult age groups was high, the classification accuracy of elderly giant pandas was not and the system often mischaracterized elderly pandas as adults. By analyzing the distribution of database samples, we speculated that the data imbalance may lead to the poor classification accuracy of older pandas. To address the problem of imbalanced data of giant pandas, we adopt cost‐sensitive re‐weighting methods to assign different weights to the samples to adjust their importance, considering that the resampling method tends to lead to the overfitting of the model. In this paper, we tried Focal Loss (Lin et al., 2017), VS Loss (Kini et al., 2021), and IB Loss (Park et al., 2021) to balance the differences between elderly group data and other categories. However, we were surprised to find that the classification accuracy of the elderly group did not improve significantly with these methods. Therefore, we further speculated that the low classification accuracy of the elderly group may also be due to the improved living conditions and the longer life span of captive giant pandas compared with wild individuals.

In the wild, the life expectancy for giant pandas is 20 years, while the oldest captive panda lived to be 38 years old (Colin, 2020; Song et al., 2006; Zhao et al., 2017). Because of this large age difference, according to the current definition of the age groups of giant pandas, it is possible that the elderly captive giant pandas have not yet shown detectable signs of aging, making the image differentiation degree not large enough. To address this, we are considering whether the age cutoff point of giant pandas should be adjusted in order to investigate the population structure of giant pandas more rationally. Due to the exceptional scarcity of field data at present, our model is currently based on images of captive giant pandas only. In addition, this experiment mainly predicts the age group of giant pandas based on their facial images, which leads to some limitations of our model, i.e., the input of the model can only use the frontal facial images of giant pandas, and if the facial angle of giant pandas in the images deviates significantly, it will inevitably lead to a decrease in the prediction accuracy of the model. In addition, the image quality of captive giant pandas is better than that of field images. Therefore, at a later stage, it is necessary to further improve the system, optimize the proposed algorithm, conduct large‐scale field dataset collection, gradually expand the dataset, solve the problems of data imbalance, age group recognition of giant panda facial images under different angles, etc. At the same time, we will further study the age group prediction of giant panda images in the wild.

As infrared cameras are widely used in conservation research fields, such as population assessment, animal resource investigation, and human–animal conflict (Carthew & Slater, 1991; Cutler & Swann, 1999; Karanth et al., 2004; Li et al., 2010; Martorello et al., 2001), they have become an increasingly powerful tool for wildlife detection around the world. A large number of images of wild animals are being collected, which can provide a reliable and sufficient data source for wildlife image recognition research. It is beneficial to the development and application of computer identification technology in the field of biology in the future, and can effectively solve the problem of inadequate utilization of monitoring data in protected areas, as well as save a lot of manpower and material resources.

At present, the combination of infrared camera data and computer graphics processing technology for further research is maturing (Brehar et al., 2021; Dai et al., 2021; Zhang & Rao, 2022; Aji et al., 2022). We expect to introduce a better recognition algorithm in the near future that will play a greater role in ecological and behavioral research of endangered and rare species, such as the giant panda, and provide a solid foundation for improving wildlife conservation and management.

## AUTHOR CONTRIBUTIONS

**Yu Qi:** Conceptualization (equal); data curation (equal); formal analysis (equal); investigation (equal); methodology (equal); project administration (equal); resources (equal); software (equal); validation (equal); visualization (equal); writing – original draft (equal). **Han Su:** Conceptualization (equal); formal analysis (equal); funding acquisition (equal); investigation (equal); methodology (equal); project administration (equal); resources (equal); supervision (equal); validation (equal); writing – review and editing (equal). **Rong Hou:** Conceptualization (equal); data curation (equal); funding acquisition (supporting); project administration (equal). **Hang‐Xing Zang:** Data curation (equal); formal analysis (equal); investigation (equal). **Peng Liu:** Data curation (equal); resources (equal); validation (equal). **Mengnan He:** Data curation (equal); resources (equal). **Ping Xu:** Data curation (equal); resources (equal). **Zhihe Zhang:** Data curation (equal); resources (equal). **Peng Chen:** Data curation (equal); funding acquisition (supporting); project administration (equal); resources (equal); writing – review and editing (equal).

## CONFLICT OF INTEREST

None of the authors have any conflict of interest to declare.

## ETHICAL APPROVAL AND CONSENT TO PARTICIPATE

The methods, use of materials, and all experimental procedures involving animals were approved by the Institutional Animal Care and Use Committee of the Chengdu Research Base of Giant Panda Breeding protocol (IACUC. 2018023). All methods were performed in accordance with the relevant guidelines and regulations under the Law of the People's Republic of China.

## Data Availability

The data that support the findings of this study are openly available in “Image data set based on the age of giant pandas” at https://doi.org/10.5061/dryad.m63xsj43n.
